# Abstracta

**Published:** 1884-07

**Authors:** 


### Abstracta.

MATTHEW ARNOLD may be a great man, but we fail to find his picture on any brand of soap.—*Phil. Call.*

Doctor—"There, get that prescription filled and take a tablespoonful three times a day before meals." Pauper patient—"But, doctor. I don't get but one meal in two days."—*Texas Siftings.*

IF you will sit down and wait, yung man, at least one haff ov the good things ov life will at sum time eddy around near yu, while the more yu chase them the more they will break into a run.—*Josh Billings.*

IN CLOVER.—He promised to cleave to her, and when they went to the theatre and he came back between the acts with a piece of cork in his whiskers, she knew from the fragrance he exhaled that he had clove.—*Druggist.*

AN English temperance society has offered prizes amounting to $6,000 for some non-intoxicating drink that will be a substitute for beer. It would first be in order, we think, to devise some non-culpable inquity that would be a substitute for sin.—*Burlington Free Press.*

It is said that the tooth manufacture of the United States amounts to 10,000,000 teeth per annum. There are twelve manufactories, but one of them, founded in 1864, makes half the number. The value of these teeth is a million dollars. The materials of which they are composed are feldspar, kaolin, and rock crystal.

SPUTA OF PHTHISICAL PATIENTS, after alternate drying and moistening, still contains bacilli, and can be successfuly used in inoculating animals. M Vignal concludes that even such sputa in the streets or in apartments might be agents of contagion to persons predisposed, in whom the baccilli would find favorable soil.—*Med. and Surg. Reporter.*

THE DOSE OF QUININE.—Professors Bartholow and Da Costa agree that an antipyretic dose of quinine is not less than five grains every two hours until four doses are taken, or else thirty grains in two or three doses close together. The former believes a small dose of morphine given with quinine is the best thing to counteract the unpleasant cerebral symptoms of the latter.

In the neigboring town of New Britain there is a factory for the production of quassia cups. The chips and shavings in the cup factory were thrown away or burned, until some of the lager-beer brewers discovered that they were available in the place of hops for lager beer; then a demand arose for them, until now the proprietor of the shop is making more money out of his chips and shavings than he is making out of his cups.—*Hartford Times.*

POISONOUS JEQUIRITY PUNCTURE.—Jequirity seeds rolled, when wet, into little needles, called "suis," and fixed lightly into a wooden handle, are used to poison cattle, in the Chamar tribe of India. An animal is stabbed with one of these and the point allowed to remain in the tissue. The animal dies in a few hours from the enormous amount of micrococci and bacteria which form in the blood. Man-poisoning has occurred by similar puncture.—*Brit. Med. Journal.*

GERMS and bacilli are rather the carriers of disease than its producers. The disease carried is probably some semi-organized ferment, like the health-giving ferments, pepsine, pancreatine, etc., or more like the products of decomposition called ptomaines. In the living blood a fibrin ferment may be rapidly liberated, which will deteriorate the blood cells and lead to septicæmia. The immediate future study of the causes of disease lies in the separation of the coincident phenomena from the real cause, and in the identification of these semi-organized ferments.—*Med. and Surg. Reporter.*

JEQUIRITY AS A POISON.—The use of jequirity in India is not of recent date, according to Ceuter. The part it plays in that country is quite as mysterious as fatal. Cases of poisoning occured in India quite often, which for a long time were very difficult of explanation. Ceuter lifts the veil, at the same time giving the mode of applying the poison. A few jequirity seeds, which are deprived of their shell, are soaked in water, then placed in Madar milk for twenty-four hours, crushed in a mortar, and rolled out into a small needle. This needle, dried in the sun and thus hardened, is thrust into the enemy's skin, who will most surely die three days afterwards· Chemical researches are incapable of establishing the presence of the poison.—*St. Louis Druggist.*

M. BREMOND has made, on sailors, soldiers, and officers, from fourteen to twenty-six years of age, a large number of experiments showing that lethargy, catalepsy and somnambulism can be produced in healthy, non-hysterical people, and that these phenomena are preceded by a peculiar state of fascination. The period of fascination is characterized by a sudden increase in the frequency of the pulse. One-third of the young men experimented upon manifested one or more of the above mentioned symptoms.—*British Medical Journal.*

---

NO SUPPURATION WITHOUT MICO-ORGANISMS. —The relation of the micro-organisms found in pus with the process of suppuration itself have been carefully investigated during the last two years, and several important papers have been published on the subject, especially in Germany. The experiments consisted generally in subcutaneous injections of sterilized irritating fluids or in the introduction under the skin of aseptic solid bodies. The results, however, have been contradictory. M. Strauss, from experiments made in M. Pasteur's laboratory, has arrived at the opinion that the introduction of irritating substances under the skin causes suppuration when micro organisms have gained access at the same time, but that simple plastic inflammation is set up when all precautions have been taken to exclude them.—*British Medical Journal.*

---

THE editor of the *Indianapolis News* is a strong upholder of our American pie. He writes the following, which we wish was so :

The English can mix up a mess of meat, potatoes, and dough a foot across and six inches deep, bake it, and when it is cold dig it out in chunks and eat it. They called that "pie," and it is allowable to eat that sort of thing—a mess of indigestible unpalatable stuff, as witness Sam Weller's testimony to the "weal pie," that it was the seasonin' as does it." But when we propose to eat a thin crust, fresh baked, with fruit, making it perfectly digestible, then the army of dudes and the funny paragraphists of the newspaper must set up a moan at the provincial ignorance which would do so. It is time this sort of nuisance should expend itself. Pie is an American dish, it is palatable, comestible, and digestible. Pie is the perfection of a lunch. Pie is healthful. Pie is good. If that be treason, make the most of it. Long live pie.

ORAL PATHOLOGY.—A red line on the gums, with fetor and metalic taste, indicates pyalism; a blue line, lead poisoning; great sponginess, with sloughing and great fetor, scurvy; a red line about the teeth and along the gums, periostitis; purple gums and purulent discharge, necrosis; gums hot, red, swollen, very tense, phlegmon; gums inflamed and soft, with fluctuation, alveolar abcess; swollen gums, fetid discharge, mucous patches, shallow ulcers under the tongue, eroded palate, eruption of mourh, skin and scalp, gums everted, fetid matter from necks of teeth, syphilis ; a white-coated tongue, denotes febrile disturbance; a brown, moist tongue, indigestion; a brown, dry tongue, depression, blood poisoning, typhoid fever; a red, moist tongue, feebleness, exhaustion; a red, dry tongue, inflammatory fever; a red, glazed tongue, general fever, loss of digestion; a tremulous, moist and flabby tongue, feebleness, nervousness; a glazed tongue, with blue appearance, tertiary syphilis. —*Independent Practitioner.*

---

The *Lancet*, in a recent number, commenting on the death of Cetewayo, which is supposed to have been due to "fatty degeneration" of the heart, says the medical evidence is not conclusive, but it is a fair presumption that death may have been caused as suggested. This conclusion must not, however, be hastily drawn from the fact that Cetewayo was a prodigal feeder. The popular error that "fatty degeneration" of the heart is the same thing as an accumulation of fat around that organ is one which ought to be corrected, as every practitioner, of course, knows how to correct it. When muscular tissue degenerates, as it may do from any one of many morbid causes, fat is deposited interstitially in the place of the atrophied organic structure. As a matter of physiological or pathological fact, "fatty degeneration" is quite as likely to occur in a thin subject as in one that is obese, and voracious feeding has nothing to do with "fatty degeneration," though in a certain proportion of instances it may have something to do with the accumulation of deposits of fat around organs and in the interspaces of muscle fasciculi. We ought to keep the discrimination of matters which are likely to be confounded always clear. Many conditions besides large eating determine the presence of fat, and "fatty degeneration" is, as we have said, something altogether apart from, and in no way to be confounded with fatness.— *Thera Gazette.*

